# Pancreatic pseudocyst, gastric outlet obstruction, superior mesenteric artery syndrome and renal vein entrapment syndrome in groove pancreatitis: a case report

**DOI:** 10.1093/gastro/goae079

**Published:** 2024-07-31

**Authors:** Amanda N Myles, Porsha Okiye, Ghida Akhdar, Raheem Robertson, Dylan Hewlett

**Affiliations:** Division of Internal Medicine, Department of Medicine, Piedmont Athens Regional Medical Center, Athens, GA, USA; Divisin of General Internal Medicine, Department of Medicine, Medical College of Georgia at Augusta University, Augusta University Medical Center, GA, USA; Division of Internal Medicine, Department of Medicine, Piedmont Athens Regional Medical Center, Athens, GA, USA; Division of Internal Medicine, Department of Medicine, Piedmont Athens Regional Medical Center, Athens, GA, USA; Division of Internal Medicine, Department of Medicine, Piedmont Athens Regional Medical Center, Athens, GA, USA

## Introduction

Groove pancreatitis (GP), an extraordinarily rare form of chronic pancreatitis, affects the groove between the head of the pancreas, second part of the duodenum, and common bile duct. GP is caused by altered pancreatic secretions in the pancreatic head resulting in surrounding inflammation [1]. GP is usually diagnosed in men from 40 to 50 years of age with a history of significant alcohol abuse, though the specific etiology, pathogenesis, and incidence of GP is unknown [2]. Two studies found the incidence of GP to be 19.5% and 24.4% [3, 4]. Early distinction of GP is essential as the condition can simulate or mask pancreatic adenocarcinoma. Subsequently, failure to recognize this entity may lead to a missed diagnosis of pancreatic or duodenal malignancy. Furthermore, the natural progression of GP remains incompletely understood, displaying a broad range of severity. Surgery is preferred for symptomatic patients following conservative treatment [5]. Management involves a multidisciplinary team including dietitians, pain management, primary care providers, radiologists, gastroenterologists, and surgeons.

Despite GP being well-documented in literature, the linkage of symptoms in this patient’s case with superior mesenteric artery (SMA) syndrome, pseudocyst development, and renal vein entrapment syndrome, also known as “nutcracker syndrome,” is significant. The interplay of these conditions appears to be correlated, offering a rare but valuable contribution to existing research and enriching international literature. To note, the incidence of pseudocysts in chronic pancreatitis is around 20%–40% [6]. Specifically, there appear to be no reported cases describing these four conditions simultaneously.

## Case report

A 38-year-old male with gastroesophageal reflux disease, alcoholic pancreatitis, and a known duodenal fluid collection/pseudocyst (3.4 × 3.3 × 2.6 cm shown on computed tomography (CT) 5 days prior) presented with increasing mid-abdominal pain, nausea, and diarrhea after consuming alcohol and an unknown 30-pound weight loss over the prior 2–3-month period. The patient was admitted to the medicine floor for medical and surgical management.

**Figure 1.**
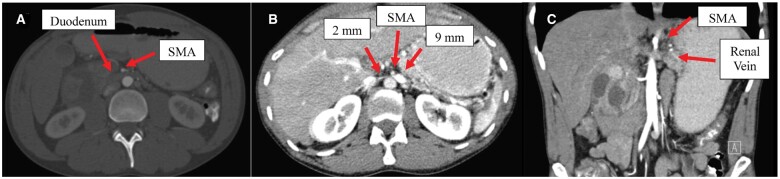
Image of superior mesenteric artery syndrome and two images of renal vein entrapment syndrome. (**A**) Contrast is not easily passing through into the second and third portion of duodenum. The third portion the duodenum does appear narrowed as it extends under the superior mesenteric artery (SMA). (**B**) This axial CT image shows atypical venous drainage of the left kidney with appearance of a nutcracker syndrome. In this view, the renal vein to the left of the SMA appears dilated measuring up to 9 mm. The renal vein more medial to the SMA is thin and strand-like measuring 2 mm. This could be a hemodynamically significant stenosis. (**C**) This CT coronal slice is another view of nutcracker syndrome evident in the patient.

The CT abdomen on admission (day 1) showed that the fluid collection had enlarged from the previous CT, measuring 4.7 × 4.0 × 3.3 cm, and appeared to be within the wall of the duodenum and common bile duct while narrowing the descending duodenum. There was distension of the stomach and duodenum to the level of the SMA, indicative of SMA syndrome as seen in Figure 1A. CT incidentally revealed a coexisting nutcracker phenomenon, the extrinsic compression of the left renal vein by the SMA anteriorly (Figure 1B and C).

A CT abdomen/pelvis on day 2 showed a pancreatic pseudocyst had ruptured into the second portion of the duodenal wall causing a narrowed aortomesenteric angle, likely SMA syndrome for which a nasogastric tube was inserted the next day, which provided emesis resolution.

On day 6, magnetic resonance imaging showed focal prominence of the pancreatic head was seen in keeping with the diagnosis of GP.

On day 7, gastroenterologist performed an esophagogastroduodenoscopy, which showed an obstructing bulge on the duodenal wall, and an endoscopic ultrasound (EUS) was performed for aspiration of a large periduodenal collection. The nasogastric tube in place was passed beyond the previously obstructed point and anchored. A repeat CT abdomen was scheduled in 4 weeks with possible nasogastric tube removal. However, the nasogastric tube was removed 3 days following discharge due to patient intolerance.

During the patient’s initial hospital course, the patient consistently asked for pain medication, for which scheduled and as needed narcotics, ondansetron, and pantoprazole were given. Following pseudocyst drainage, he endorsed a minimal ease in discomfort. He was sent home with Creon supplementations, vitamins, a short course of opioids, and arranged outpatient substance abuse rehabilitation.

The patient then returned to the Emergency Department 1 month following discharge with return of symptoms and similar sized fluid collection in the right upper abdomen measuring up to 4.5 cm. Repeat EUS was notable for small fluid collection (2.8 × 0.8 cm) around the second portion of the duodenum, which was too small for intervention. The patient has since had four additional Emergency Department visits for acute or chronic pancreatitis secondary to alcohol consumption.

## Discussion

This atypical patient is unlike those commonly described in literature with GP. The patient was below the normal age range for those with GP and had significant complications including an encircling pseudocyst with severe gastric outlet obstruction. Loss of mesenteric fat pad with BMI of 17 kg/m^2^ likely contributed to his SMA syndrome and nutcracker syndrome. Though it appears that these additional findings interplay and relate to his GP rather than are incidental, independent disease processes, a better understanding of the relationship between these disease etiologies allows for evidence-based treatment.

The decision to initially proceed with esophagogastroduodenoscopy and then proceed with EUS-aspiration vs either cystogastrostomy approach was based on physician preference. EUS is reported to be preferred over surgical approach as it has been associated with safety, clinical efficiency, and better visualization of extraluminal structures [7].

The primary team felt the patient’s pain was related to chronic pancreatitis as well as visceral stretch and gastric outlet obstruction from the pseudocyst. While pseudocyst drainage provided relief, a long-term pain management option would be necessitated. Besides medications and surgery, there are limited effective avenues for pain relief. A recent and increasingly popular form of pain management related to chronic pancreatitis is celiac plexus block [8, 9]. A few well-structured studies have evaluated the efficacy of this intervention in the management of pain due to chronic pancreatitis [10–12]. However, the meta-analysis in 2020 did not find statistically significant data supporting its effectiveness in relief [13].

This patient with GP and SMA syndrome, pathologies not previously reported together in literature, showcases a poorly understood condition while stressing GP as a challenging diagnosis for patients with nonspecific symptoms while highlighting the challenges in pain management. Additionally, the simultaneous occurrence of SMA syndrome and nutcracker syndrome is rare as their coexistence has only been reported in a few cases, but these syndromes should be evaluated for by radiologists as potentially shared pathophysiology impacts patient management [14, 15].

## Authors’ Contributions

All authors wrote the paper; A.N.M prepared the figure; all authors analyzed and interpreted the data; all authors revised the paper for important intellectual content. A.N.M and G.A. read and approved the final manuscript.
